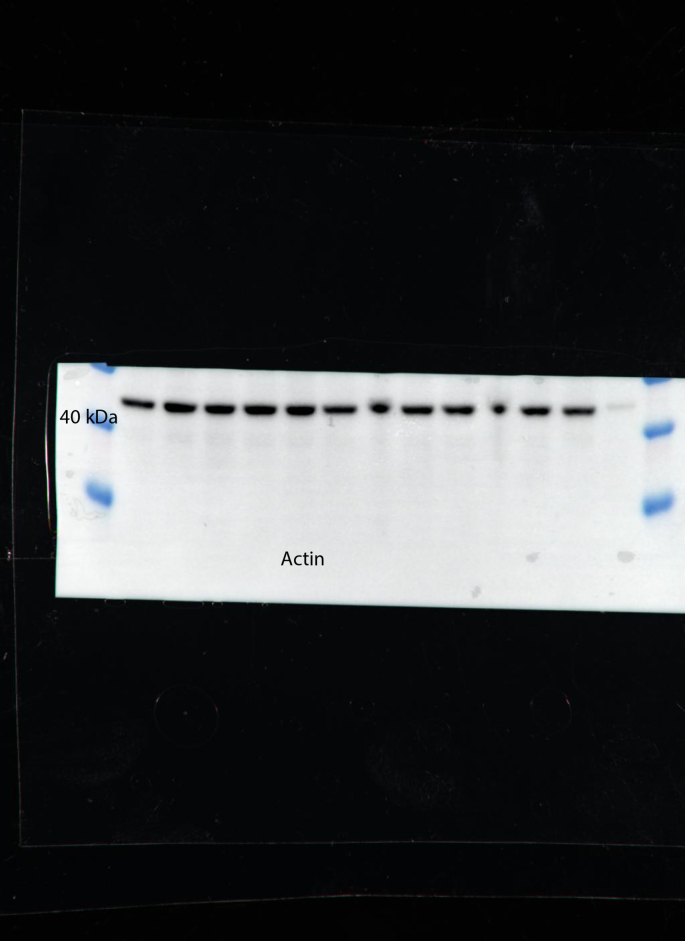# T lymphocyte-derived IFN-γ facilitates breast cancer cells to pass the blood-brain barrier: An *in vitro* study corroborating translational data

**DOI:** 10.1016/j.heliyon.2024.e36598

**Published:** 2024-08-20

**Authors:** Rute M.S.M. Pedrosa, Johan M. Kros, Benjamin Schrijver, Cor Berrevoets, Rute B. Marques, Casper C.H.J. van Eijck, Reno Debets, PieterJ.M. Leenen, Willem A. Dik, DanaA.M. Mustafa

**Affiliations:** a Department of Pathology, The Tumor Immuno-Pathology Laboratory, Erasmus University Medical Center, Rotterdam, the Netherlands; b Department of Immunology, Erasmus University Medical Center, Rotterdam, the Netherlands; c Department of Medical Oncology, Laboratory of Tumor Immunology, Erasmus University Medical Center, Rotterdam, the Netherlands; d Department of Urology, Erasmus University Medical Center, Rotterdam, the Netherlands; e Department of Surgery, Erasmus University Medical Center, Rotterdam, the Netherlands

**Keywords:** Brain, Metastasis, Breast cancer, Interferon-γ, Blood-brain barrier, Immune response, T lymphocytes

## Abstract

The appearance of brain metastasis is the most serious complication of breast cancer with mostly fatal outcomes. To reach the brain, tumor cells need to pass the blood-brain barrier (BBB). The molecular mechanisms underlying penetration of the BBB are largely unknown. Previously we found that tumor-infiltrating T lymphocytes enhance the development of brain metastasis of estrogen receptor-negative (ER-) breast cancer. In the current study, we investigate the contribution of T lymphocytes and the IFN-γ pathway in enabling breast cancer cells to pass the *in vitro* BBB. CD8^+^ cells display the strongest stimulatory effect on breast cancer cell passage. We show that inhibition of the IFN-γ receptor in MDA-MB-231 breast cancer cells, or neutralization of soluble IFN-γ, impairs the *in vitro* trespassing of breast cancer cells. Importantly, we validated our findings using gene expression data of breast cancer patients. The CXCL-9,-10,-11/CXCR3 axis, dependent on IFN-γ signaling activity, was overexpressed in primary breast cancer samples of patients who developed brain metastasis. The data support a role for T-lymphocytes and the IFN-γ pathway in the formation of brain metastasis of ER-breast cancer, and offer targets to design future therapies for preventing breast cancer cells to cross the BBB.

## 1 Introduction

The devastating formation of brain metastasis occurs in 10–16 % of breast cancer patients and is usually a late event in the natural course of the disease. The median survival following cerebral seeding is very limited, ranging from only a few months to two years [1,2]. Among all breast cancer subtypes, human epidermal growth factor receptor 2 (HER2)-positive - and triple-negative (estrogen- and progesterone receptors- and HER2 protein-negative) breast cancers have a higher risk of developing brain metastases [[3], [4], [5], [6]]. The development of cerebral metastases depends primarily on the successful penetration of the blood-brain barrier (BBB) by circulating tumor cells. The underlying mechanisms and molecular pathways utilized by the breast cancer cells to cross the BBB are largely unknown, although several genes and pathways were associated with this event [7].

Tumor cells need to take the hurdles of brain endothelial cells with their specific tight junctions and basal membrane substance, and the astrocytic end-feet that cover the brain capillaries. The expression of cell surface receptors and adhesion molecules like integrins and selectins make the tumor cells dock on to the endothelial cells [8]. Inflammatory factors like chemokines influence the passage through the tight junctions between the endothelial cells [9] and the role of the microglial cells, representing resident immune cells, is topic of current investigations [10]. Only a small proportion of tumor cells will become invasive and high level of CD44 expression along with low CD24, in combination with activity of transcription factors like OCT3/4, are prerequisites for this event [11,12]. In addition, the tumor cells upregulate the expression of IL-1α, IL-6, IL-8 and urokinase plasminogen activator, in order to break through the basal membrane of the endothelial cells and the astrocytes [13]. Tumor lineage-specific cross talk and molecular pathways contribute to the complexity of the mechanisms involved in passing the BBB.

Previously, by comparing gene expression profiles of primary breast cancer samples of women with and without brain metastasis, we found that T lymphocyte-related genes are associated with brain seeding [14]. We demonstrated that ER-breast cancer cells that were co-cultured with activated T cells displayed an increased propensity to cross an *in vitro* blood-brain barrier (BBB). Moreover, we found specific upregulation of guanylate-binding protein 1 (GBP1) in the tumor cells crossing the BBB and we also demonstrated that GBP-1 was overexpressed in the primary breast cancer samples of those women who had developed brain metastases [14]. GBP1 is a GTPase in the dynamin superfamily and a downstream target of the interferon-gamma (IFN-γ) pathway. T lymphocytes are known to secrete IFN-γ upon activation and interaction with cancer cells [15,16]. In mouse models on experimental allergic encephalitis IFN-γ stabilizes the integrity of the BBB by upregulation of tight junction proteins between endothelial cells, while expression of the IFN-γ receptor prevents the brain from inflammation [17]. These data contradict the finding of the specific upregulation of IFN-γ in reovirus encephalitis causing BBB leakage due to junctional disorganization [18]. The effects seem to be dependent on stage of inflammation and the engagement of specific immune cell types like microglia. The effects of IFN-γ on tumor cells, particularly in relation to the BBB, have remained unexplored.

So far, neither the particular subset of T cells, nor the T cell derived cytokine(s) that contribute most to the enhanced transmigration of the breast cancer cells through the BBB, have been identified. The aim of the present study was to reveal which T cell subset(s) facilitate BBB transmigration of MDA-MB-231 breast cancer cells most, and verify the involvement of IFN-γ and its signaling pathway in this event.

## 2 Materials and methods

### 2.1 T lymphocyte fluorescence-activated flowcytometric cell sorting

Total T lymphocytes (CD45^+^ CD3^+^), CD4^+^ T lymphocytes (CD45^+^ CD3^+^ CD4^+^) and CD8^+^ T lymphocytes (CD45^+^ CD3^+^ CD8^+^) were sorted using FACSAria III™ (BD Biosciences) from peripheral blood mononuclear cells (PBMC) collected from three healthy donors. (approved by the local medical ethical committee; MEC-2016-202). Peripheral blood samples were collected in lithium heparin tubes (BD Biosciences) and the PBMCs were isolated by using Ficoll-based density separation. PBMCs were incubated for 15 min at room temperature (RT) with an antibody mixture consisting of CD45-PerCP (clone: 2D1; BD Biosciences), CD3-FITC (SK7; BD Biosciences), CD4-PB (RPA-T4; BD Biosciences), CD8-PE-Cy7 (SFCI21Thy2D3; Beckman Coulter). The distinct T cell populations were isolated (purity >95 %) and transferred to RPMI-Hepes media, supplemented with 6 % human serum albumin (HSA, Sigma-Aldrich), 1 % penicillin-streptomycin (P/S) and 1 μg/ml IL-2 (360 IU/mL, Chiron, Amsterdam, The Netherlands) within 1 h.

### 2.2 Activation and Expansion of T lymphocytes

The sorted T cell fractions were cultured in RPMI supplemented with 10 % fetal bovine serum (FBS, ScienCell) and 1 % antibiotics (penicillin and streptomycin) and stimulated overnight with a commercially available phorbol 12-myristate 13-acetate (PMA) and ionomycin cell stimulation cocktail (CSC, eBioscience™, Invitrogen), according to manufacturer's instructions. Bulk T lymphocytes were activated as previously described [14]. In short, PBMCs were co-cultured with gamma-irradiated (40 Gy) allogeneic PBMCs, Epstein-Barr Virus (EBV)-transformed B-lymphoblast cell lines BSM (also known as GM06821, GLC^neg^/HLA-A2^pos^) and APD (also known as GM06817, EAD^neg^/HLA-A1^pos^) cells, in combination with Phytohemagglutinin-L (PHA-L, Sigma) and IL-2 in a 96-well flat-bottomed plate for 6–7 days at 37 °C in a humidified incubator with 5 % CO_2_. After incubation, cells were harvested, centrifuged, and cultured in RPMI-Hepes medium supplemented with HSA and IL-2. All T cell activations were performed prior to co-culturing experiments.

### 2.3 Cell line culture procedure

Human astrocytes (ScienCell) were cultured in astrocyte medium (AM, ScienCell) supplemented with 1 % astrocyte growth factors (AGS, ScienCell), 2 % FBS and 1 % P/S. Human umbilical vein endothelial cells (HUVECs, ScienCell) were cultured in endothelial cell medium (ECM, ScienCell) supplemented with 1 % endothelial cell growth factors (ECGS, ScienCell), 5 % FBS and 1 % P/S. Human astrocytes and HUVECs were used between passage 2 and 5. MDA-MB-231 breast cancer cell line (ER-, human breast cancer cell line derived from a patient with metastases in brain and other organs [19]), was used and cultured in RPMI-1640 with L-glutamine (BioWhittaker®) medium supplemented with 10 % FBS and 1 % P/S.

### 2.4 Construction of the *in vitro* blood-brain-barrier (BBB) model

Details of the BBB *in vitro* model were described previously [14,20].

### 2.5 Measuring the transmigration of breast cancer cells through the *in vitro* BBB

Because we previously found similar effects of the T cells on 3 different breast cancer cell lines [21] and the MDA-MB-231 line is most commonly used, we selected this line for the present investigations. To investigate the influence of T lymphocytes on the ability of the breast cancer cells to cross the BBB, MDA-MB-231 cells were co-cultured for 72 h with previously activated T lymphocytes (sorted subsets or CD3^+^ bulk T lymphocytes), at 37 °C in a humidified incubator with 5 % CO_2_. As control, MDA-MB-231 cells were used alone, or in the presence of cell stimulation cocktail. To investigate the influence of the T-lymphocyte-secreted factors in the absence of the T cells, the conditioned medium (CM) of activated T cells was added to the cancer cells for 3 consecutive days. The co-culture ratios of the cancer cells/T lymphocytes, and of volume RPMI/CM of T lymphocytes was 3:1. After 3 days of co-culture the T cells were removed by three-step PBS washings. The breast cancer cells were trypsinized and labeled with 5 μM CFMDA cell tracker green (Invitrogen) in serum-free medium, for 45 min, at 37 °C. The breast cancer cells were collected, PBS-washed and re-suspended in full-serum medium. Approximately 1.5 × 10^5^ cancer cells were seeded in the upper chamber of the BBB model and incubated overnight at 37 °C in a humidified incubator with 5 % CO_2_. Subsequently, the transwells were removed and the living cancer cells adhered to the bottom of the 24-well chamber were nuclear-stained with Hoechst 33342 (Invitrogen) and recorded by confocal microscopy. Confocal images were obtained using a Zeiss LSM510 confocal laser-scanning microscope equipped with a 488 nm argon-laser, a 405 nm Diode and a Plan-Neofluar 20 × objective with NA 0.5 (Zeiss, Oberkochen, Germany). Pictures were submitted to ImageJ software version 1.49S (http://www.fiji.sc) and used to calculate the number of cells per mm^2^. A schematic overview of the experimental procedures is shown in Fig. 1.

**Fig. 1:**
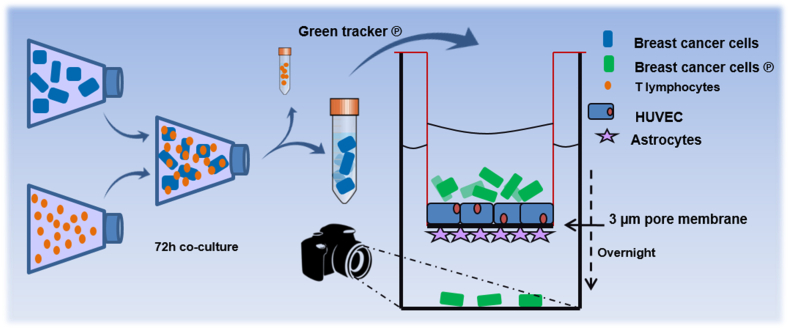
Schematic presentation of the *in vitro* experiments using the BBB model. Legends Fig. 1: Scheme of *in vitro* experimental design. MDA-MB-231 breast cancer cells were co-cultured with activated T lymphocytes. Following removal of the T lymphocytes the breast cancer cells were labeled with fluorescent green dye and added to the upper chamber of the BBB model. Transmigration of the cells was monitored using a confocal laser-scanning microscope (Zeiss LSM510) equipped with a 488 nm Argon-laser and a Plan-Neofuar 20 × objective with NA 0.5 (Zeiss, Oberkochen, Germany). Images were made with a pixelsize of 0.9 μm. and transferred to the ImageJ software version 1.49S (http://www.fji.sc) for calculation of the number of cells per mm2.

### 2.6 IFN-γ signaling pathway blocking and inhibition experiments

The IFN-γ receptor consists of IFNGR1 (CD119 or subunit α), responsible for binding ligand in a species-specific manner, and IFNGR2, (AF-1 or subunit β), required for induction of biologic responses. Because IFNGR2 is constitutively expressed at low levels and is up-regulated by external stimuli [22,23], this subunit is used for monitoring successful blockade of the IFN-γ pathway [22,23] (Supplemental Fig. 1). To block the IFN-γ receptor, MDA-MB-231 breast cancer cells were pre-incubated for 1h with a concentration range (0.01–10 μg/ml) of human IFNGR1/CD119 monoclonal antibody (clone 92101; R&D Systems) or 10 μg/ml Mouse IgG1 isotype control (clone 11711; R&D Systems). Subsequently, the cancer cells were incubated overnight with CM from activated bulk T lymphocytes. In order to study the effect of soluble IFN-γ (sIFN-γ), anti-hIFN-γ-IgA (clone H7WM120; InvivoGen, San Diego, CA, USA) or human IgA2 isotype control (anti-βGal, clone T9C6; InvivoGen, San Diego, CA, USA) was added to the CM of activated bulk T lymphocytes for 1h, prior to overnight incubation with the cancer cells. Culture supernatants were collected and MDA-MB-231 breast cancer cells were harvested for functional studies and protein Western blot analysis. IFN-γ signaling induces phosphorylation of two STAT1 residues: tyrosine 701 (Y701), which facilitates dimerization, nuclear translocation and DNA binding; and Serine 727 (S727) enabeling maximal STAT1 transcriptional activity [24]. Serine 727 phosphorilation grants nearly 80 % of IFN-γ-induced transcriptional activity [25].

Supernatants from: a) non-activated and overnight activated T lymphocyte subsets, before and after 72h incubation time with MDA-MB-231 breast cancer cells; b) 5, 10, 20 or 30 min or one, two, four, six or 24h MDA-MB-231 breast cancer cell culture, alone or incubated with CM from activated bulk T lymphocytes, or after co-culture with T lymphocyte; c) 5, 15, 30 and 60 min, and 24h MDA-MB-231 breast cancer cell culture, alone or incubated with 10 ng/ml rIFN-γ, were collected and IFN-γ levels were measured (DuoSet ELISA, R&D Systems, Minneapolis, MN, USA). ELISA was performed following the manufacturer's instructions, and each sample was measured in duplo.

### 2.7 Reverse transcription-polymerase chain reaction (RT-PCR)

Total RNA was extracted from previously snap-frozen cell pellets using the RNeasy Plus Micro kit (Qiagen), quantitated using NanoDrop 1000 (NanoDrop Technologies) and reversely transcribed into cDNA using the RevertAid H-Minus first-strand cDNA synthesis kit (Thermo Scientific) according to the manufacturer's protocol. Quantitative real-time PCR was performed using TaqMan Master Mix (Applied Biosystems) on the 7500 Real-Time PCR system, v.2.3 (Applied Biosystems). The following commercially available exon-spanning TaqMan Gene Expression Assays (Applied Biosystems) were used: IFN-γ, exon 3–4 (Hs00989291_m1), HPRT1, exon 2–3 (Hs02800695_m1) and HMBS, exon 13–14 (Hs00609296_g1). HPRT1 and HMBS were used as reference genes. The relative quantification of target gene expression was performed using the 2^−ΔΔCt^ comparative method and the threshold cycle value was defined by the point at which there was a statistically significant detectable increase in fluorescence.

### 2.8 Western blot

Cells were washed twice with ice-cold PBS and scraped in RIPA Buffer (ThermoScientific, Rockford, USA). A protease and phosphatase inhibitor cocktail (Halt™, ThermoScientific, Rockford, USA) was added to RIPA lysis buffer before use. Cell lysates were centrifuged (1000g for 15 min). The protein content of the cleared lysates was determined (Pierce™ BCA Protein Assay Kit; ThermoScientific, Rockford, USA). Protein lysates were boiled in SDS-sample buffer and separated by SDS-PAGE (12.5 % acrylamide). Proteins were blotted onto nitrocellulose membranes (BIO- RAD; Bio- Rad Laboratories, Hercules, CA, USA) and probed with the following antibodies: phospho-STAT1 (Ser727, PSM1, 1:1000, Thermo Fisher Scientific), total-STAT1 (SM1, 1:1000; Thermo Fisher Scientific), IFN-γ R2 (1:2000, R&D Systems), GBP1 (1B1, 1:200, Santa Cruz) and actin (C-2, 1:1000, Santa Cruz). Visualization was achieved by the chemiluminescence kit (BM Chemiluminescence Western Blotting Substrate (POD), Roche) and a maximum sensitivity substrate (SuperSignal West Femto Maximum Sensitivity Substrate, Thermo Scientific), according to the manufacturers’ instructions. Western blot images were acquired and analyzed through a digital Western blot scanner Amersham™ Imager 600 (GE Healthcare Bio-Sciences AB, Uppsala, Sweden). Densities of proteins of interest were measured and normalized towards the structural control protein actin or, in the case of phosphorylated proteins, to its matched total protein.

### 2.9 Re-analysis of the gene expression profile data

To check upregulations of additional genes involved in the IFN-γ pathway, particularly the CXCR3/XCL9, CXCL10 and CXCL11 axis, the previously generated gene expression data of primary breast cancers from patients with (n = 13) and without (n = 9) brain metastasis were analyzed [14]. This axis is known to be involved in differentiation, migration, and tumor infiltration of immune cells, directly or indirectly affecting metastatic behavior [26]. Morphological assessment, RNA expression profiling and relevant clinical data regarding this discovery cohort were previously provided [14].

### 2.10 Statistical analysis

Prism 5.0 (GraphPad Software) was used to perform statistical tests. A two-tailed Student's *t*-test was used to determine differences in the sample means. Data are presented as means ± SD. In all statistical analyses p-values of <0.05 were considered statistically significant. Unless otherwise stated, all *in vitro* experiments were repeated independently three times.

## 3 Results

### 3.1 Activated CD8^+^ T lymphocytes most strongly stimulate MDA-MB-231 breast cancer cells to pass the BBB

All cell stimulating cocktail (CSC)-activated T lymphocyte fractions increased the ability of MDA-MB-231 breast cancer cells to cross the *in vitro* BBB. However, the CD8^+^ T lymphocyte fraction stimulated the BBB-passage of the breast cancer cells more than the CD4^+^ T-lymphocyte fraction did (p < 0.05). Addition of CSC to the breast cancer cells without activated T lymphocytes did not result in an increased BBB passage (Fig. 2a and b ).

**Fig. 2:**
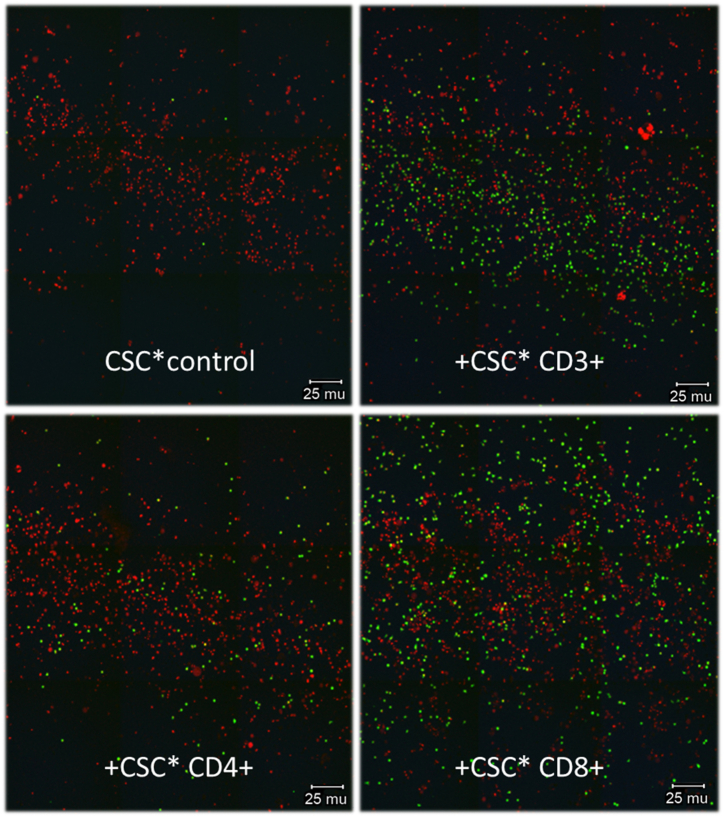

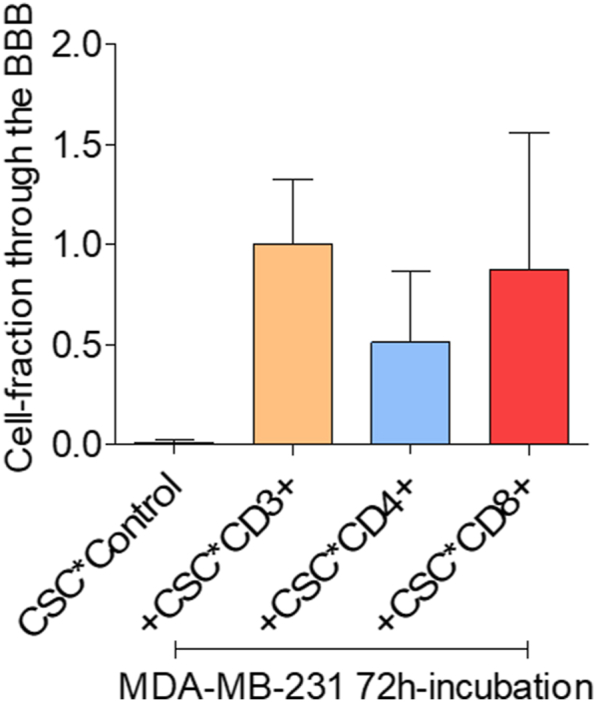
aConfocal images taken of the breast cancer cells that reached the bottom of the 24-well chambers by crossing the *in vitro* BBB. Legends Fig. 2a: Confocal images were obtained using a Zeiss LSM510 confocal laser-scanning microscope equipped with a 488 nm Argon-laser and a Plan-Neofuar 20 × objective with NA 0.5 (Zeiss, Oberkochen, Germany). Images were made with a pixelsize of 0.9 μm. Pictures were submitted to ImageJ software version 1.49S (http://www.fji.sc) and used to calculate the number of cells per mm^2^. Cancer cells that were incubated with cell stimulation cocktail (CSC) only did not cross the *in vitro* BBB (control, upper panel left). The number of cancer cells that crossed the *in vitro* BBB were larger following co-culture with CD8^+^ than with CD4^+^ T cells (p < 0.05). (Green dots: breast cancer cells; red dots: nuclei of cells other than breast cancer cells; living cancer cells nuclear-stained with Hoechst 33342). Legends Fig. 2b: The fractions were calibrated to the fraction of tumor cells co-cultured with activated CD3^+^ T cells. Mean values ± SD; data from 3 independent experiments. CSC = cell stimulating cocktail. Fig. 2bMean-percentages breast cancer cells that passed the *in vitro* BBB after incubation with PMA (ctr), and after co-culture with CSC-activated T lymphocyte subtypes.

### 3.2 T cell secreted IFN-γ

Concentrations of soluble IFN-γ (sIFN-γ) in the media of the CSC activated fractions of T cells were determined. Upon CSC activation and co-culture with the breast cancer cells, the respective T cell fractions secreted variable concentrations of IFN-γ in the media. No IFN-γ was detectable in the media of CSC-exposed breast cancer cells. The CD8^+^ cells secreted approximately four-fold higher levels of IFN-γ than CD4^+^ cells did (p < 0.001, Fig. 2a). The IFN-γ levels dropped significantly 72 h after culturing the breast cancer cells in the CM of the CD3^+^, CD4^+^ and CD8^+^ T lymphocytes (Fig. 3a).

**Fig. 3:**
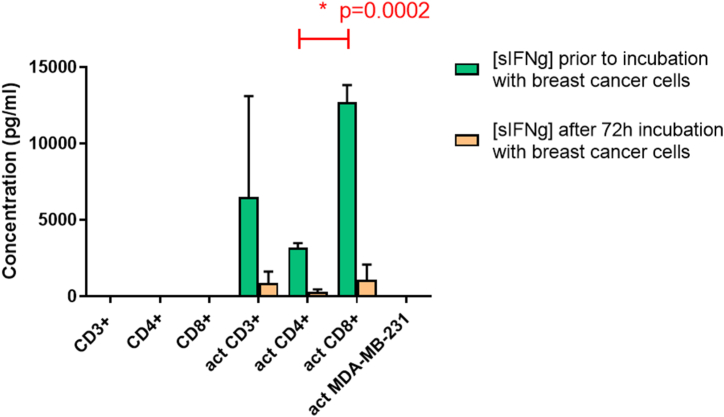

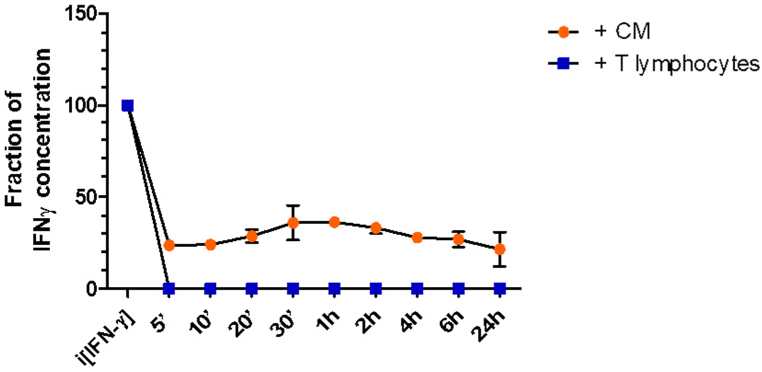
a: Concentrations of sIFN-γ in the conditioned media of the respective T cell fractions before and after adding the MDA-MB-231 breast cancer cells Legends to Fig. 3a: No sIFN-γ was detected in the media of non-activated T cells (left three lanes). Activated T cells secreted sIFN-γ and CD8^+^ T cells secreted higher concentrations of sIFN-γ than CD4^+^ cells did (p < 0.001; green bars). There is significant decrease in concentrations of sIFN-γ in the CM following 72 h of incubation with the tumor cells (orange bars). After 72 h incubation with the cancer cells no significant differences in IFN-γ levels were present between the CM of CD8^+^ or CD4^+^ cells (orange bars). Activation of MDA-MB-231 breast cancer cells did not result in secretion of IFN-γ (most right column). Graph representative of two independent experiments (act = activated as described in the M&M section). Legends to Fig. 3b: Concentration curve of sIFN-γ following incubation of the cancer cells with CM of activated bulk T cells (orange dots), or co-culture with activated bulk T cells (blue blocks). Within the first 5 min a steep drop in IFN-γ is recorded for both conditions. In the presence of the T cells sIFN levels are down to undetectable levels, probably because of uptake of sIFN by the T cells. Graph representative of two independent experiments. CM = conditioned medium; (s)IFN-γ = (soluble) interferon gamma. Fig. 3b: sIFN-γ concentration curve.

In order to monitor sIFN-γ concentrations in the media over time the supernatant was collected at different time points following incubation of breast cancer cells with either activated T lymphocytes (MDA + T), or with the media conditioned by activated T lymphocytes (MDA + CM). After 5 min of co-culturing the cancer cells with the T cells sIFN-γ was no longer detectable(Fig. 3b). Upon incubation with the CM without the T cells the sIFN-γ levels dropped to approximately 20 % of the initial concentrations and did not change over 24 h (Fig. 3b).

### 3.3 IFN-γ neutralization and IFNGR1 receptor blocking in the breast cancer cells impairs passing the BBB

In order to investigate whether IFN-γ enhanced BBB passage of the MDA-MB-231 breast cancer cells we used neutralizing antibodies to the IFN-γ molecule (anti-hIFN-γ-IgA), and blocked the INF-γ receptor IFNGR1 by using a monoclonal antibody (human IFNGR1/CD119). Blocking of IFNGR1 resulted in reduction in protein expression of subunit IFNGR2 of the IFNγ receptor (Fig. 4). Blocking IFNGR1 in the breast cancer cells prior to overnight incubation with CM of activated bulk T lymphocytes resulted in a concentration-dependent decrease in passage of the tumor cells through the *in vitro* BBB (Fig. 5a). Neutralization of IFN-γ in the media of the activated bulk T lymphocytes by using anti-hIFN-γ mAb (10 μg/ml) prior to the incubation with the tumor cells also significantly reduced tumor cell passage across the *in vitro* BBB (80 %; p < 0.002, Fig. 5b).

**Fig. 4:**
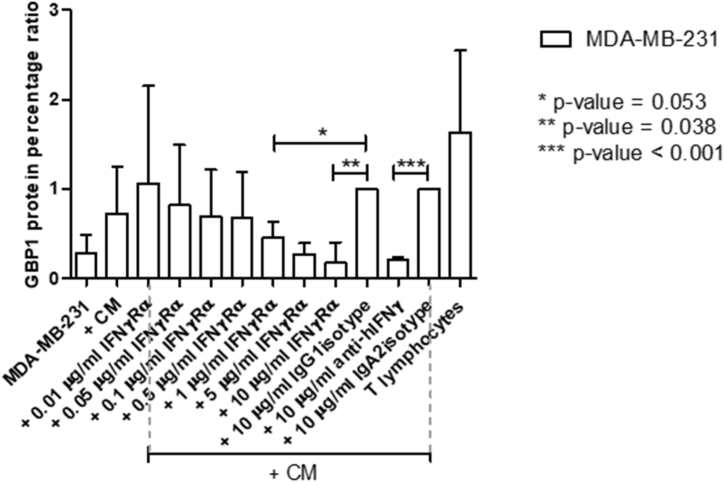
IFNGR2 protein expression is reduced in MDA-MB-231 breast cancer cells after IFNGR1 blocking. Legends to Fig. 4: IFNGR2 expression levels after blocking IFNGR1 in MDA-MB-231 breast cancer cells in a dose dependent manner. Significant protein reduction is evident when using concentrations of IFNGR1 ≥ 1 μg/ml. IFNGR2 expression was calculated after background subtraction and normalization against actin. IFNGR2 level-fractions are relative to 10 μg/ml matched hIgG1 isotype control. Bars are mean values with SD. Ctr = MDA-MB-231 control cells; CM = conditioned medium; IgG1 = anti hIgG1 isotype control. Graph is representative of two independent Western blot analyses.

**Fig. 5:**
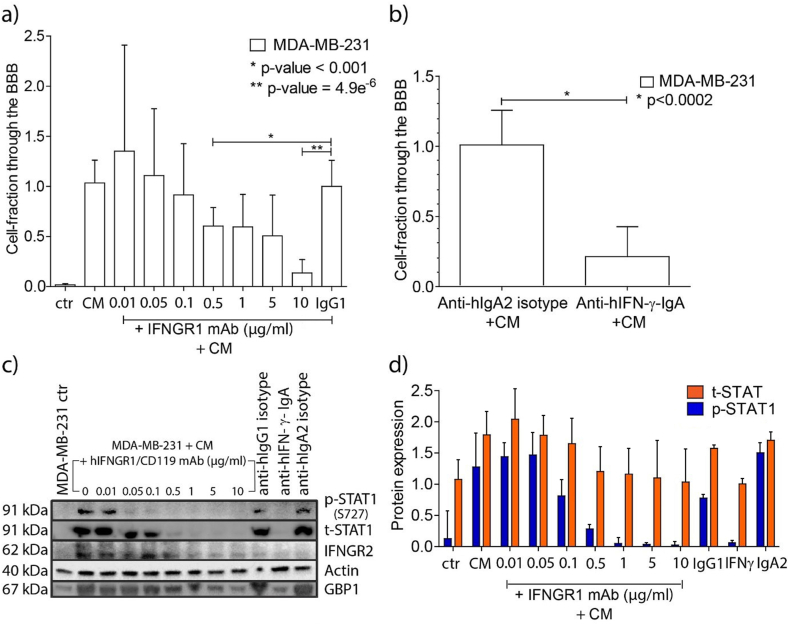
Blocking of the IFN-γ pathway in MDA-MB-231 breast cancer cells impairs passage through the BBB model. Legends to Fig. 5: a) Fractions of transmigrated cancer cells through the *in vitro* BBB model after 1 h incubation with increasing concentrations of human IFNGR1/CD119 mAb and subsequent overnight incubation with equal amounts of CM of activated bulk T cells. The fractions are relative to 10 μg/ml IgG1 isotype control. (ctr, MDA-MB-231 control cells; CM, conditioned medium; IgG1, hIgG1 isotype control). Graph representative of three independent functional experiments. b) Fractions of transmigrated cancer cells after neutralizing the sIFN-γ in the media of activated T cells. The fractions are relative to 10 μg/ml anti-IgA2 control. Graph representative of two independent experiments. Human IgA2 isotype control was used as control. c) Western blots of phosphorylated STAT1 (p-STAT1), total STAT1 (t-STAT1), IFNGR2 and GBP1 protein expression levels in MDA-MB-231 samples after blocking IFNGR1 (experiment described in a.) or after neutralizing the sIFN-γ in the media of activated T cells. Actin was used as loading control for both membranes. A concentration-dependent reduction of p-STAT1, t-STAT1, IFNGR2 and GBP1 proteins was observed by using increased concentrations of human IFNGR1/CD119 monoclonal antibody. Western blots representative of two independent experiments. For non-adjusted blots see Supplementary Files 1–5. d) Normalized expression of t-STAT1 and p-STAT1 in the cancer cells following concentration-dependent IFNGR1 blocking in the cancer cells, or after neutralizing the sIFN-γ in the media of activated T cells. The level of p-STAT1 is increased by the addition of CM to the breast cancer cells and step-wise curtailed until it matched the p-STAT1 level observed in MDA-MB-231 control cells, synchronous with IFN-γ pathway blocking. t-STAT1 and p-STAT1 expression levels were calculated in the cancer cells after background deduction and normalization against actin and normalized t-STAT, respectively. (ctr, MDA-MB-231 control cells; CM, conditioned medium; IgG1, anti hIgG1 isotype control; IFN-γ, anti-hIFN-γ-IgA; IgA2, anti hIgA2 isotype control. Graph representative of two independent Western blots).

To ascertain the effects of blocking the IFN-γ pathway in the cancer cells, activation of the downstream signaling molecule STAT1 was monitored by measuring phosphorylated STAT1 (p-STAT1) related to total STAT1 (t-STAT1) (Fig. 5c). Concentration-dependent reduction of p-STAT1 was observed along with increasing concentrations of the blocking antibody to IFNGR2 (Fig. 5c); graphic presentation Fig. 5d). In addition, we monitored the expression of GBP1 that was found to be upregulated in breast cancer cells upon co-culturing with activated T lymphocytes in our previous work [14]. Expression of both IFNGR2 and GBP1 was reduced upon IFNGR1 blockage (Fig. 5c and d; Supplementary materials Fig. 5c files 1–5).

### 3.4 IFN-γ, CXCL9, -10, −11 and CXCR3 are overexpressed in primary breast cancer samples of patients who developed brain metastasis

**Fig. 6:**
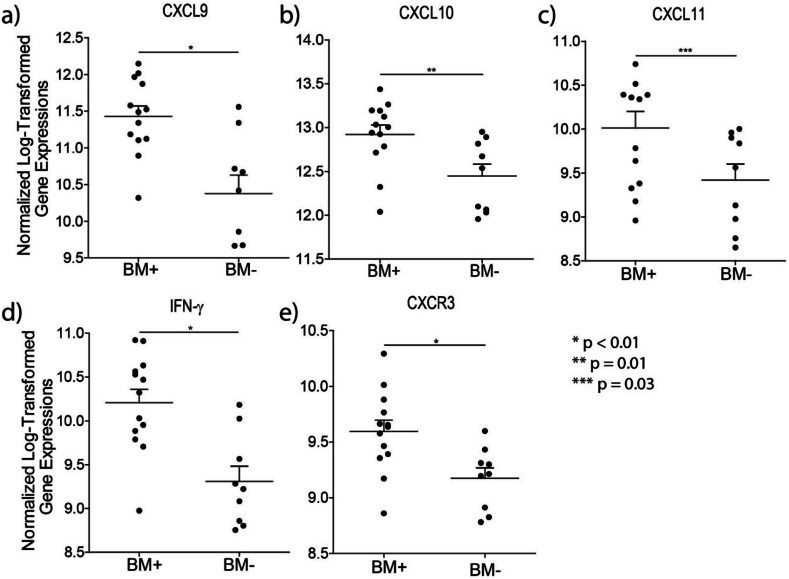
Overexpression of IFN-γ-inducible chemokines in primary breast cancer samples of patients who developed brain metastasis. Legend Fig. 6: a-e: CXCL9, -10, −11/CXCR3 axis and IFN-γ are overexpressed in primary breast cancer samples of patients who developed brain metastasis. Solid lines indicate the mean gene expression and the error bars the SEM. Normalized log 2-transformed gene expression data were used to calculate significance by the two-tailed unpaired Student's *t*-test. BM+, primary breast cancer sample of patients who developed brain metastasis; BM-, primary breast cancer sample of patients who developed metastasis at sites other than brain.

By analyzing the gene expression data of primary breast cancer samples of patients who developed brain metastasis (n = 13) or metastasis to organs other than the brain (n = 9), the expression levels of the IFN-γ-inducible chemokines CXCL9, -10, −11, and their receptor CXCR3, were found to be significantly higher in the primary breast cancers from patients who developed brain metastasis (Fig. 6a, b, c and e). In line with the current findings, the expression of the IFN-γ gene was found to be significantly higher in the breast cancers that developed brain metastasis (1.86-fold, p < 0.001, Fig. 6d).

## 4 Discussion

Based on expressional differences between tumors with and without brain metastases we discovered a relation between T lymphocytes invading primary breast cancers and their potency of seeding to brain. In additional *in vitro* and *in vivo* studies we found that T cells affect the passage of breast cancer cells through a modelled BBB [8]. We also described the specific upregulation of GBP1 in the tumor cells as consequence of T cell interactions. In general, the effects of an inflammatory infiltrate on tumor progression and prognosis of the patients are numerous and complex [[27], [28], [29]]. Although the influence of T cell infiltrates on clinical behavior of breast cancers has been noticed in the past, a specific brain metastasis-promoting effect was not described previously [[30], [31], [32]]. Two important questions, namely the particular T cell subset that promotes the brain seeding most, and the role of interferon-γ, being a prominent secreted cytokine of T cells, remained unanswered.

In the present study we found that CD8^+^ T lymphocytes affect the passage of MDA-MB-231 breast cancer cells through the BBB strongest, and that T-lymphocyte-derived IFN-γ plays a prominent role in this phenomenon. It has long been recognized that CD8^+^ T lymphocytes may not purely function as cytotoxic killer cells, but also have important regulatory functions through differential cytokine production [[33], [34], [35]]. CD8^+^ lymphocytes are known to be more efficient producers of IFN-γ than CD4^+^ T lymphocytes [36], a finding corroborated by the significant higher IFN-γ levels secreted in the media of activated CD8^+^ T cells in this study. CD8 binds MHC class I molecules and upon activation CD8^+^ lymphocytes will secrete higher levels of IFN-γ than CD4^+^ lymphocytes, which bind MHC class II molecules. It seems that the CD8^+^ T cell cytotoxic actions require higher expressional levels of IFN-γ than the helper functions displayed by the CD4^+^ lymphocytes.

Following incubation of CM of the activated T cells with the breast cancer cells, the levels of IFN-γ lowered quickly, indicative of utilization by the cancer cells. The upregulation of (p)STAT1 and GBP1 demonstrates the intracellular implications. The differences in residual levels of sIFN-γ following co-culturing with the activated T cells, or incubation with their CM, may be a sequel of re-uptake by an upregulation of the IFN receptor on the activated T cells [37].

To test whether an active IFN-γ signaling pathway in MDA-MB-231 breast cancer cells would be crucial for their successful passage across the *in vitro* BBB, we impaired the IFN-γ pathway in breast cancer cells by either blocking the IFN-γ receptor on the MDA-MB-231 breast cancer cells, or by neutralizing the sIFN-γ in the CM of activated T lymphocytes. Both interventions strongly reduced the ability of the breast cancer cells to cross the BBB. In order to validate the blocking effect on the IFN-γ pathway we monitored phosphorylation of the downstream signaling protein STAT1. Our data show that at a concentration of ∼0.5 μg/ml IFNGR1 mAb, the protein expression levels of both total STAT1 and phosphorylated STAT1 (p-STAT1) decreased to levels comparable to those observed in unstimulated MDA-MB-231 breast cancer cells. The findings seem to align with data from patho-biology: blocking of IFN-γ is beneficial to reduce metastasis formation of tumors of various lineages [38,39]. Specifically, the IFN-γ signaling pathway plays a role in growth and metastasis of triple-negative breast cancer [40]. IFN-γ is a potent molecule that regulates the expression of over 100 genes acting in various pathways [41]. A major cluster of those genes includes chemokines and their receptors, which - among other functions - are involved in the recruitment and directional migration of specific cell types [42]. In previous work we found upregulation of GBP1 in breast cancer cells following co-culturing with activated T lymphocytes [14]. The expression of GBP1 is under control of IFN-γ [43,44] and blocking of the IFN-γ pathway with inhibitor doses ≥5 μg/ml significantly decreased GBP1 expression (Fig. 5c and d) and significantly decreased passage of breast cancer cells through our *in vitro* BBB model (Fig. 5a). These data fit in with the assumption that GBP1 expression, dependent on IFN-γ, is linked with increased motility of the cells [45,46]. Blocking of the IFN-γ pathway did, however, not result in a complete transmigration arrest (∼80 % maximal achieved inhibition). Although blockade of the IFN-γ receptor with an anti-IFNGR1 or an anti–IFN–γ antibody completely abolished STAT-1 phosphorylation induced by culture medium from activated T cells (Fig. 5c and d), it did not result in complete transmigration arrest (∼80 % maximal achieved inhibition) (Fig. 5a and b). Therefore, we conclude that the IFN-γ pathway is not exclusively responsible for effective BBB-passage, and that pathways other than that of IFN-γ play additional roles in facilitating transmigration of breast cancer cells across the BBB. Obviously, elucidation of this additional pathways is a necessity for successful prevention of brain metastasis in patients.

In order to validate the overexpression of IFN-γ and IFN-γ inducible chemokines known to be involved in tumor progression and metastasis [26], expressional levels of the CXCR3/CXCL9, CXCL10, CXCL11 axis were scrutinized in ER-breast cancer samples of women with metastatic disease, with or without brain seeding. By checking on the expressional levels of these molecules in the gene expression profiles of women suffering from metastasized ER-breast cancers w/wo brain involvement we found significant overexpression in the tumors that had metastasized to brain. The expression of the chemokines CXCL9, CXCL10 and CXCL11, as well as their receptor CXCR3 is induced by IFN-γ levels. CXCL9, also referred to as monokine induced by IFN-γ (MIG) and induced by IFN-γ [21], appeared to be the highest overexpressed molecule among the CXCL9, -10, −11/CXCR3 axis found in the primary breast cancer samples that developed brain metastasis (Fig. 6). CXCL9 acts as a homing chemokine and is constitutively expressed in brain [21]. In addition, the CXCL9,-10,-11/CXCR3 signaling axis takes part in intracellular communication and plays a crucial role during the pathogenesis of various central nervous system (CNS) diseases [[47], [48], [49], [50]]. Human brain endothelial cells and astrocytes modulate IFN-γ-inducible chemokines and are involved in the invasion of the CNS by immune cells [21] and likely also circulating cancer cells. CXCL9 is overexpressed in human astrocytes compared to human brain endothelial cells [21], suggestive of a continuous overexpression of IFN-γ-inducible chemokines on the brain-side of the BBB, and establishing a concentration gradient that attracking metastasizing cells. The cross-talk between the tumor cells and brain cells in this context warrants further studies.

## 5 Conclusions

We proved the involvement of T lymphocyte-mediated activation of IFN-γ signaling in breast cancer cells facilitating their migration through an *in vitro* BBB model. Our results offer directions for further investigations of the mechanisms underlying the formation of brain metastasis, which will be helpfull in future therapy design for the prevention of cancer cells crossing the BBB in patients with breast cancers.

## Funding

This work was supported by the Department of Pathology of the Erasmus University Medical Center and by Support Casper Foundation: www.supportcasper.nl.

## Ethical review and approval, informed consent

Were not applicable for this study because it did not involve humans or animals.

## Data availability statement

Has data associated with your study been deposited into a publicly available repository?

No. Data are available on request.

## Ethical approval

The experiments involved human tumor tissues, for which use approval was obtained by the Medical Ethical Committee of the Erasmus Medical University, communication code MEC-2017-1060. Informed consent was obtained from all patients involved concerning the use of their biological materials and clinical and laboratory data. The study and experiments were conducted in accordance with all regulations of sound laboratory experimenting.

## Co-authors’ approval

All authors have seen the contents of the paper and have approved the Manuscript for submission.

## Justification of authors’ contributions

All authors have contributed sufficiently to warrant co-authorship.

## Submission procedure

(None of) this work is currently under review elsewhere, or was published previously.

## CRediT authorship contribution statement

**Rute M.S.M. Pedrosa:** Writing – original draft, Project administration, Investigation, Formal analysis, Data curation, Conceptualization. **Johan M. Kros:** Writing – original draft, Supervision, Resources, Project administration, Methodology, Investigation, Funding acquisition, Formal analysis, Conceptualization. **Benjamin Schrijver:** Methodology, Investigation. **Cor Berrevoets:** Resources, Methodology, Investigation. **Rute B. Marques:** Resources, Methodology, Investigation, Formal analysis. **Casper C.H.J. van Eijck:** Investigation, Funding acquisition, Formal analysis. **Reno Debets:** Methodology, Investigation, Formal analysis. **PieterJ.M. Leenen:** Resources, Methodology, Investigation, Formal analysis. **Willem A. Dik:** Writing – original draft, Validation, Resources, Methodology, Investigation, Formal analysis. **DanaA.M. Mustafa:** Writing – original draft, Supervision, Project administration, Methodology, Investigation, Funding acquisition, Formal analysis, Data curation, Conceptualization.

## Declaration of competing interest

None of the authors has any conflict of interest concerning the contents of this work.

## Figures and Tables

**figs1:**
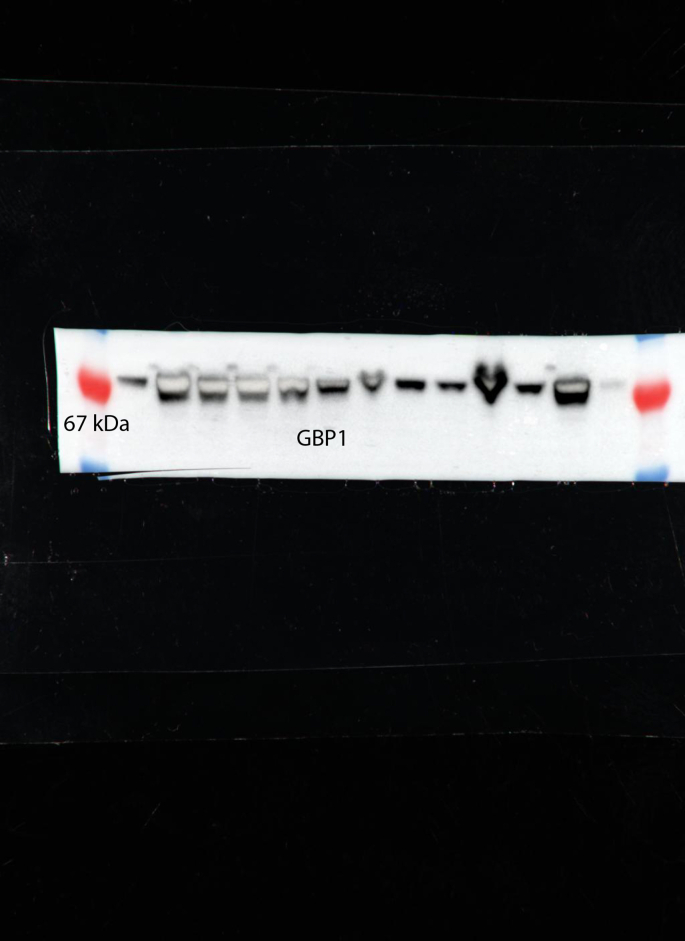


**figs2:**
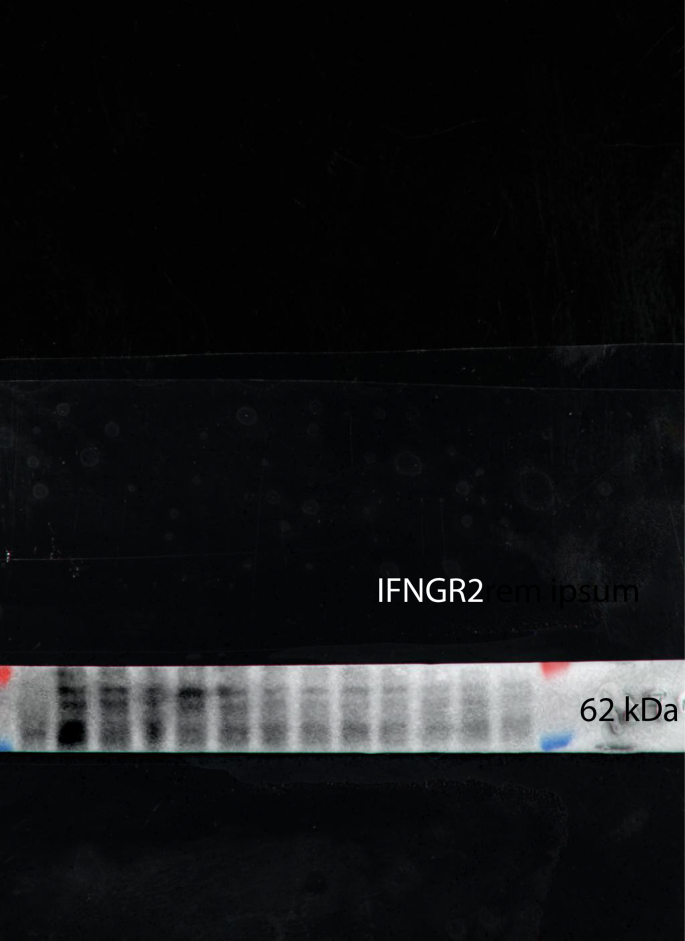


**figs3:**
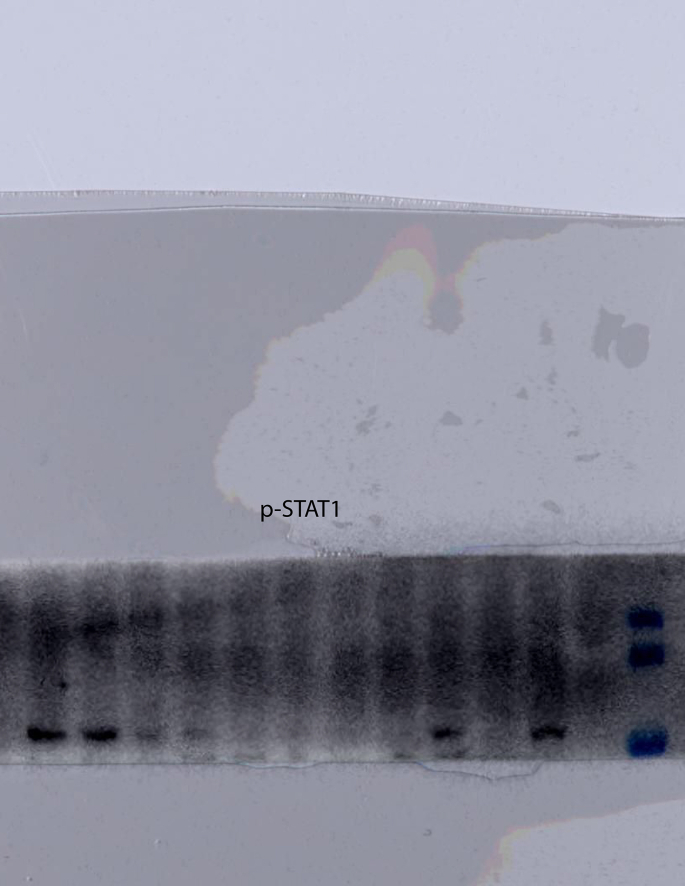


**figs4:**
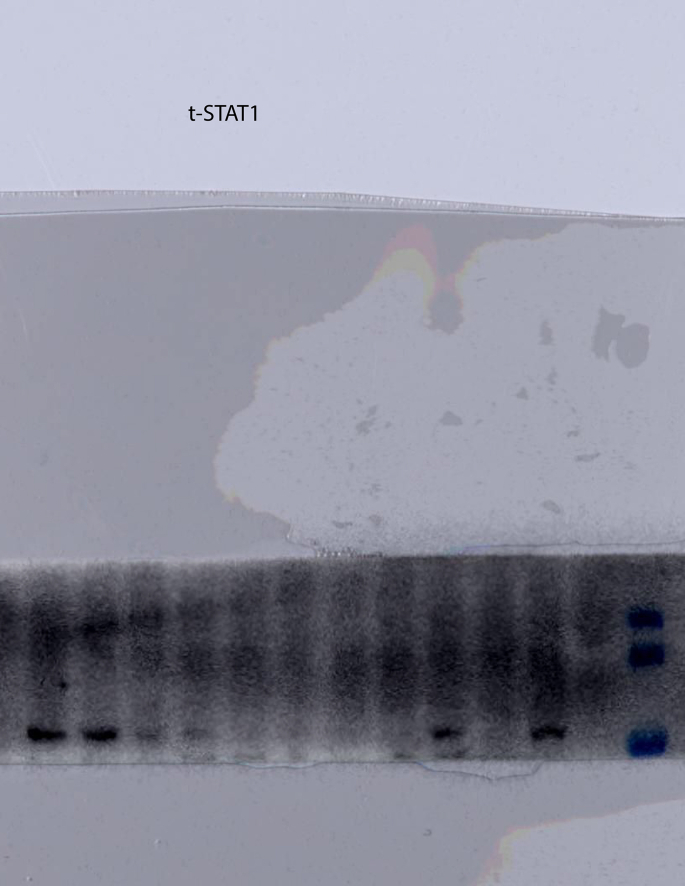


**figs5:**